# Tumour cells engineered to secrete interleukin-15 augment anti-tumour immune responses in vivo

**DOI:** 10.1038/sj.bjc.6690538

**Published:** 1999-07

**Authors:** S Hazama, T Noma, F Wang, N Iizuka, Y Ogura, K Yoshimura, E Inoguchi, M Hakozaki, K Hirose, T Suzuki, M Oka

**Affiliations:** Departments of Surgery II and Biochemistry II, Yamaguchi University School of Medicine, 1144 Kogushi, Ube, Yamaguchi, 755, Japan; Biomedical Research Institute, Kureha Chemical Industry, 3-26-2 Hyakunin-cho, Shinjuku-ku, Tokyo, 169, Japan

**Keywords:** IL-15, tumour immunity, vaccination, in vivo animal models, gene therapy

## Abstract

We examined the effect of interleukin-15 (IL-15) gene transfer into tumour cells on the host's anti-tumour response. In BALB/c mice IL-15 producing Meth-A cells (Meth-A/IL-15) underwent complete rejection, in a response characterized by massive infiltration of CD4^+^ T-cells and neutrophils. In contrast, Meth-A cells transfected with vector alone (Meth-A/Neo) grew rapidly. Moreover, rechallenged parental cells also were rejected in association with CD8^+^ T-cell infiltration. However, in nude mice there was no drastic difference between Meth-A/IL-15 and Meth-A/Neo cells. These results demonstrate that IL-15-secreting tumour cells can stimulate local and systemic T-cell-dependent immunity and therefore may have a potential role in cancer therapy. © 1999 Cancer Research Campaign


*British Journal of Cancer* (1999) **80**(9), 1420–1426
© 1999 Cancer Research Campaign
Article no. bjoc.1999.0538

### Tumour cells engineered to secrete interleukin-15 augment anti-tumour immune responses in vivo

**S Hazama[1], T Noma[2], F Wang[1], N Iizuka[1], Y Ogura[1], K Yoshimura[1], E Inoguchi[3], M Hakozaki[3], K Hirose[3], T Suzuki[1] and M Oka[1]**

Departments of [1]Surgery II and [2]Biochemistry II, Yamaguchi University School of Medicine, 1144 Kogushi, Ube, Yamaguchi 755, Japan; [3]Biomedical Research Institute, Kureha Chemical Industry, 3-26-2 Hyakunin-cho, Shinjuku-ku, Tokyo 169, Japan

**Summary** We examined the effect of interleukin-15 (IL-15) gene transfer into tumour cells on the host's anti-tumour response. In BALB/c mice IL-15 producing Meth-A cells (Meth-A/IL-15) underwent complete rejection, in a response characterized by massive infiltration of CD4+ T-cells and neutrophils. In contrast, Meth-A cells transfected with vector alone (Meth-A/Neo) grew rapidly. Moreover, rechallenged parental cells also were rejected in association with CD8+ T-cell infiltration. However, in nude mice there was no drastic difference between Meth-A/IL-15 and Meth-A/Neo cells. These results demonstrate that IL-15-secreting tumour cells can stimulate local and systemic T-cell-dependent immunity and therefore may have a potential role in cancer therapy.

**Keywords:** IL-15; tumour immunity; vaccination; in vivo animal models; gene therapy

Interleukin (IL)-15 is a novel cytokine that uses β- and γ-chains of the IL-2 receptor (R) for signal transduction and shares biologic activities with IL-2 (Giri et al, 1994; Grabstein et al, 1994). IL-15 has been reported to activate and stimulate the proliferation of T- and B-lymphocytes (Armitage et al, 1995; Mori et al, 1996). It also stimulates the proliferation of natural killer (NK) cells (Carson et al, 1996) and facilitates the induction of cytolytic effector cells including cytotoxic T-lymphocyte (CTL) and lymphokine-activated killer (LAK) cells (Gamero et al, 1995; Lewko et al, 1995; Munger et al, 1995). These properties suggest that IL-15, like IL-2, may be of value in cancer treatment. Unlike IL-2, however, IL-15 is produced by a wide variety of tissues and cells that include activated monocytes/macrophages, skeletal muscle, kidney, epithelial cells, activated endothelial cells and placenta (Grabstein et al, 1994; Bamford et al, 1996). Moreover, the IL-15R employs a unique α-chain which has a higher affinity and broader tissue distribution than the IL-2R α-chain (Andeson et al, 1995; Giri et al, 1995). These differences in the expression pattern of IL-15 and its receptor compared to the IL-2/IL-2R system suggest unique in vivo roles for IL-15.

Transfection of various cytokine genes [IL-2, IL-4, IL-6, IL-7, IL-8, IL-10, IL-12, IL-13, tumour necrosis factor (TNF)-α, interferon (IFN)-γ, granulocyte-macrophage colony stimulating factor (GM-CSF), granulocyte-colony stimulating factor (G-CSF) and macrophage inflammatory protein (MIP)-1α (Colombo and Forni, 1994; Hirose et al, 1995; Lebel-Binay, et al, 1995; Zitvogel et al, 1995)] into tumour cells results in inhibition of tumour growth. This effect is mediated through the infiltration of T-lymphocytes, macrophages and/or polymorphonuclear leucocytes into the tumour sites. These studies have shown the primary effects of cytokine release as well as effects seen with the induction of secondary cytokines (Colombo and Forni, 1994). However, there are no studies that have examined transfection of the IL-15 gene into animal tumour cells, and its effects on the anti-tumour immune response in vivo.

In this study we transfected tumour cells with an IL-15 expression vector and examined the response to the IL-15-secreting tumour cells in both BALB/c and BALB/c *nu/nu* mice with defective T-lymphocyte-mediated immunity.

### MATERIALS AND METHODS

### RT-PCR and primers

RNA isolation and reverse transcription polymerase chain reaction (RT-PCR) were carried out as previously described with some modifications (Iizuka et al, 1995). Briefly, cells ($5 \times 10^6$) were solubilized in 1 mL of TRIzol reagent (Life Technologies, Grand Island, NY, USA) and total cellular RNA was isolated according to the manufacturer's instructions. One microlitre of total RNA (1 µg) was added to 19 µl of RT-mixture (Takara, Ohtsu, Japan). After mixing, the samples were incubated at 30°C for 10 min, 55°C for 30 min, 95°C for 5 min and 4°C for at least 5 min. PCR-mixture (80 µl) (Takara) containing 100 nM primers was added to the RT products. PCR cycling parameters were as follows for the isolation of human IL-15 (hIl-15) cDNA and for the selection of hIL-15 mRNA-expressing clones: denaturation at 94°C for 1 min, annealing at 60°C for 1 min, and extension at 72°C for 2 min. PCR amplification of the IL-15 and β-actin genes utilized 40 and 28 cycles respectively.

The primer sequences were as follows – IL-15-5′ primer: 5′-GATCCCAAGCTTCCGTGGCTTTGAGTAATGAG-3′ and 3′ primer: 5′-CGCGCCTCTAGAGCAATCAAGAAGTGTTGATG-3′ (Grabstein et al, 1994); β-actin-5′ primer: 5′-ATGGATGATGATATCGCCGCGCT-3′ and 3′ primer: 5′-CGGACTCGTCATACTCCTGCTTG-3′ (Tokunaga et al, 1986). The expected sizes of the PCR products were 532 bp for IL-15 and 1224 bp for β-actin.

*Received 8 September 1997*
*Revised 16 September 1998*
*Accepted 12 January 1999*

*Correspondence to:* S Hazama

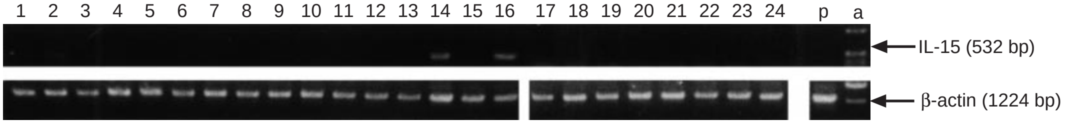

**Figure 1** Expression of IL-15 and β-actin in G418 resistant clones and parental Meth-A cells (lane p). Lane a is the 1 kb DNA ladder. Lanes 1–18 represent IL-15-transfected clones and lanes 19–24 vector-only clones. IL-15 mRNA was detected in clones 2, 14 and 16. Arrows indicate IL-15 and β-actin mRNA

To determine whether Meth-A cells used produce murine IL-15, other primers for human and murine IL-15 were prepared using the sequences without homology between human and murine IL-15 mRNA. PCR cycling parameters were the same as described above. PCR amplification of human and murine IL-15 was 28 cycles, and β-actin was 24 cycles. The primer sequences were as follows – human IL-15-5′ primer: 5′-GCCAACTGGGTGAATGTAATA-3′ and 3′ primer: 5′-TCAAGAAGTGTTGATGAACAT-3′; murine IL-15-5′ primer: 5′-GAGGAATACATCCATCTCGTGC-3′ and 3′ primer: 5′-GAGTCATGTTACTGTACTCATG-3′ (Nishimura et al, 1998); β-actin-5′ primer: 5′-TCGACAACGGCTCCGGCATGT-3′ and 3′ primer: 5′-GCTGATCCACATCTGCTGGAA-3′.

The expected sizes of the PCR products were 347, 296 and 1046 bp for human-IL-15, murine IL-15 and β-actin respectively. The PCR products were subjected to 1% agarose gel electrophoresis and visualized by staining with ethidium bromide.

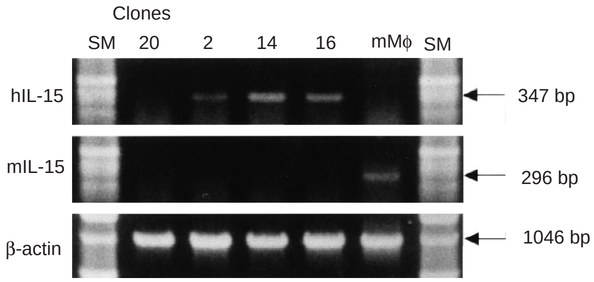

**Figure 2** Expression of human IL-15, murine IL-15 and murine β-actin in clones 2, 14, 16 and 20. SM is the 1 kb DNA ladder. Clones 2, 14 and 16 represent human IL-15 transfectants, and clone 20 vector-only transfectant. Human IL-15 mRNA was detected in clones 2, 14 and 16, whereas murine IL-15 was not detected in any transfectant. Arrows indicate IL-15 and β-actin mRNA. SM, size marker; mMϕ, lipopolysaccharide-stimulated murine macrophages; bp, base pair

### Human IL-15 cDNA expression vector

A human IL-15 cDNA was isolated by RT-PCR using total RNA purified from lipopolysaccharide-stimulated human peripheral blood mononuclear cells. The PCR fragment was cloned into a *Hin*dIII/*Xba*I site of the pGEM-4Z plasmid (Promega, Madison, WI, USA), and the nucleotide sequence was confirmed by sequence analysis. The same fragment was then recloned into a *Hin*dIII/*Xba*I site of the pRc/CMV eukaryotic expression vector (Invitrogen, San Diego, CA, USA).

### Tumour cells and transfection

The Meth-A cell line, a methylcholanthrene-induced fibrosarcoma of BALB/c origin (DeLeo et al, 1977), was maintained in RPMI-1640 medium supplemented with 10% heat-inactivated fetal calf serum, 100 U $ml^{-1}$ penicillin G and 100 g $ml^{-1}$ streptomycin. Subconfluent cultures in 100-mm petri dishes were transfected with 5 μg of hIL-15 expression plasmid or vector alone using the lipofectAMINE reagent (Life Technologies) according to the manufacturer's instructions. G418 (200 μg $ml^{-1}$) (Life Technologies) was added to the cells 48 h later. G418-resistant clones were isolated and expanded in culture medium containing 100 μg $ml^{-1}$ of G418.

### Measurement of IL-15 production by bioassay

Each transfectant as well as the parental Meth-A cells ($1 \times 10^6$ cells $ml^{-1}$) were cultured in serum-free RPMI-1640 without G418 for 24 h. The culture supernatants were collected by centrifugation for 5 min at 400 ***g*** and were concentrated using Centricon concentrators (10 000) (Grace, Danvers, MA, USA) to tenfold enrichment prior to bioassay. IL-15-dependent CTLL-2 cells (4000 cells per well) in 96-well flat microtitre plates were incubated with culture medium supplemented with $5 \times 10^{-5}$ M 2-mercaptoethanol in the presence of various concentrations of hIL-15 (Genzyme, Cambridge, MA, USA) or culture supernatant with or without anti-hIL-15 monoclonal antibody (mAb; M111, Genzyme) at various concentrations. After a 20 h incubation 50 μg of (3-(4,5-dimethylthiazol-2-yl)-2,5-diphenyl tetrazolium bromide) (MTT) (Chemikon International, Temecula, CA, USA) were added to each well, and the reaction was allowed to incubate for an additional 4 h at 37°C. Isopropanol with 0.04 N hydrochloric acid (100 μl) was then added. Colour development at a wavelength of 540 nm was monitored by an enzyme-linked immunorsorbent assay (ELISA) reader (SLT Labinstruments, Austria). The sensitivity of this bioassay was 50 pg $ml^{-1}$.

### Measurement of IL-15 production by ELISA

IL-15 levels were measured using ELISA (BioSource, Camarillo, CA, USA). In each assay a standard curve using recombinant cytokine was constructed and each sample assayed in duplicate. The sensitivity of the assay was 37 pg $ml^{-1}$.

### Animal studies

Seven-week-old female BALB/c mice and male BALB/c *nu/nu* mice were purchased from Japan SCL (Hamamatsu, Japan). Injections of Meth-A cells were carried out with freshly prepared suspensions at a concentration of $2 \times 10^7$ cells $ml^{-1}$. The total number of tumour cells injected per animal was $2 \times 10^6$. All injections were subcutaneous (s.c.) in the right lower abdominal quadrant via a 27-gauge needle. Tumour volumes were measured in $mm^3$ with a vernier caliper and determined according to the following formula: $a \times b^2 \div 2$, where a is the larger and b the smaller of the two dimensions. All animal experiments were

*© 1999 Cancer Research Campaign*

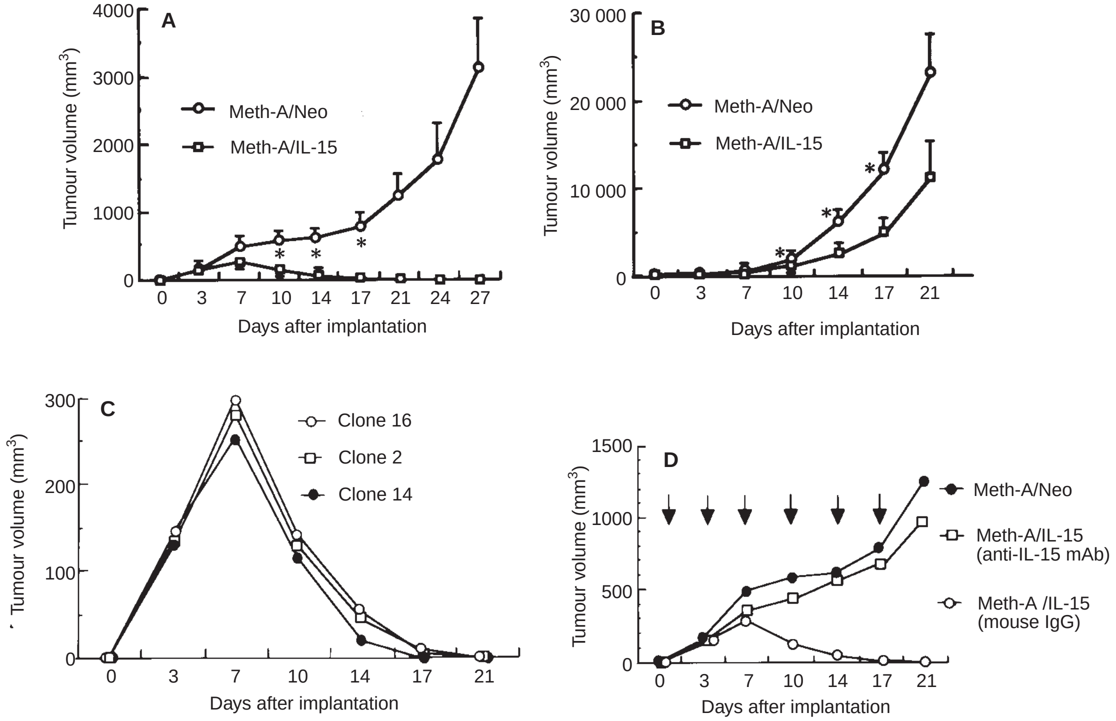

**Figure 3** In vivo growth of Meth-A/IL-15 and Meth-A/Neo cells. BALB/c mice (**A**) and BALB/c *nu/nu* mice (**B**) were implanted subcutaneously with $2 \times 10^6$ cells ($n = 10$). Tumour growth was suppressed in Meth-A/IL-15 (* $P < 0.02$). Tumour volumes are mean ± s.e.m. (**C**) In-vivo growth of three IL-15-producing clones ($n = 5$). There was no difference in the growth of clones 2, 14 and 16. These clones produced different amount of bioactive IL-15. Tumour volumes are mean. Each s.e.m. was < 10%. (**D**) The effect of anti-human IL-15 mAb on the growth of Meth-A/IL-15 in vivo. Mice were treated by intraperitoneal injection with anti-human IL-15 mAb at 50 µg per injection on days 0, 3, 7, 10, 14, 17, after tumour cell implantation ($n = 5$). Normal mouse IgG was injected into untreated mice on the same schedule ($n = 5$). Arrows indicate the days of antibody injection. The growth of Meth-A/IL-15 was augmented by the injection of anti-human IL-15 mAb. Tumour volumes are mean. Each s.e.m. was < 10%

conducted in accordance with UKCCCR Guidelines on Animal Welfare (Workman et al, 1988).

### Rechallenge with Meth-A or colon 26

Thirty days after disappearance of the initial Meth-A/IL-15 implantation, 20 mice were injected with $2 \times 10^6$ parental Meth-A cells in the previously uninjected side (left) of the lower abdominal quadrant. Colon-26 cells, a murine colon adenocarcinoma derived from BALB/c mice (Corbett et al, 1975), were also injected into both non-immunized and immunized mice (BALB/c mice that had rejected Meth-A/IL-15 cells).

### Antibody treatment in vivo

In vivo neutralization of produced hIL-15 in tumorigenesis studies was accomplished by intraperitoneal injection with a mouse anti-h IL-15 mAb (M111, Genzyme) at 50 µg per 0.2 ml per injection on days 0 (the day of tumour cell injection), 3, 7, 10, 14 and 17 after tumour cell injection. This antibody at approximately 5–10 µg $ml^{-1}$ neutralized approximately 80% of bioactivity induced by 5 ng $ml^{-1}$ IL-15 in CTLL-2 cells. Normal mouse IgG (50 µg per 0.2 ml per injection; Caltag Laboratories, Burlingame, CA, USA) was injected into untreated mice on the same schedule.

### Histologic evaluation and immunohistochemical staining

On day 7 and 14 after inoculation, the tumours were dissected, fixed in 10% neutral-buffered formalin and embedded in paraffin. Sections (4-µm) were stained with haematoxylin and eosin. For immunohistochemical staining, tissues were embedded in OCT compound (Ames Division, Miles Laboratories, Elkart, IN, USA), snap-frozen in liquid nitrogen, and stored at –80°C. Aceton-fixed 6-µm cryostat sections were blocked with goat serum and then immunostained with optimal dilution of the following rat mAbs: L3/T4 (CD4: Becton Dickinson, Franklin Lakes, NJ, USA), KT15 (CD8:Serotec, Sapporo, Japan), Mac-1 (CD11b: Caltag). The slides were then sequentially incubated with biotinated goat anti-mouse Igs (ZYMED Laboratories, San Francisco, CA, USA) and ABComplex (DAKO, Tokyo, Japan). Each incubation step lasted at least 30 min and was followed by a 10 min phosphate-buffered saline (PBS) wash. Sections were then incubated with 0.03% hydrogen peroxide and 0.06% 3,3′diaminobenzidine for 2–5 min, washed in tap water and counterstained in haematoxylin.

### Statistical analysis

Statistical analysis used the Student’s *t*-test. A value of $P < 0.05$ was considered statistically significant. Results are presented as mean ± s.e.m.

### RESULTS

### Expression of IL-15 in transfectants

Eighteen independent G-418-resistant colonies were isolated and expanded. RNA was isolated and RT-PCR performed. Three (2, 14, 16) of 18 clones expressing IL-15 mRNA were selected for

*© 1999 Cancer Research Campaign*

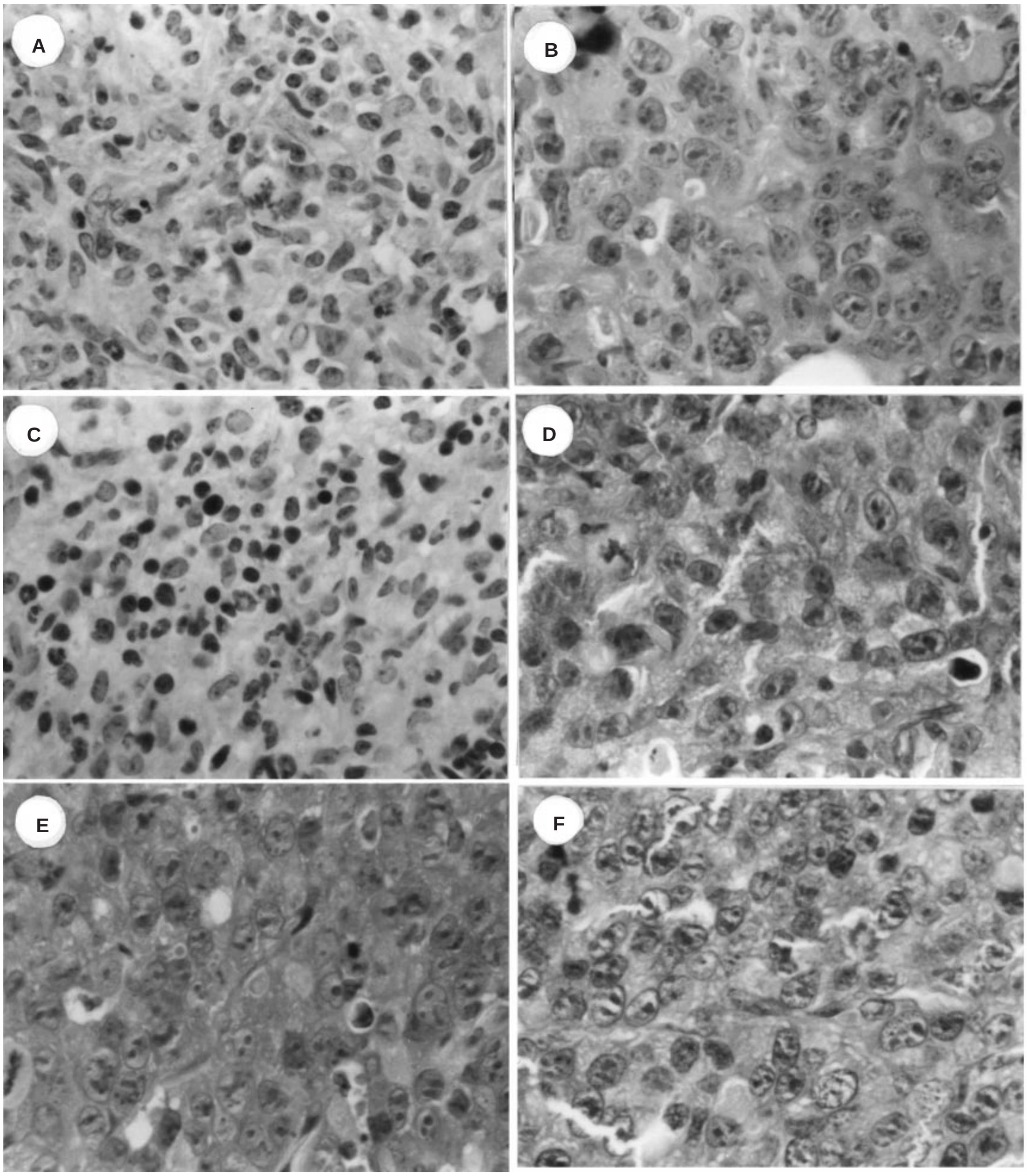

**Figure 4** Histological examination of tumour implantation sites. BALB/c mice (**A**–**D**) were implanted subcutaneously with IL-15-producing Meth-A cells (**A**, **C**) or Meth-A/Neo cells (**B**, **D**), and the injection sites were resected 7 (**A**, **B**) and 14 days (**C**, **D**) after implantation. BALB/c *nu/nu* mice (**E**, **F**) were implanted subcutaneously with IL-15 producing Meth-A cells (**E**) or Meth-A/Neo cells (**F**) and the injection sites were resected 14 days after implantation (haematoxylin and eosin, × 400)

bioassay. Endogenous IL-15 mRNA was not detected in Meth-A cells transfected with vector alone and parental cells (Figure 1). Endogenous murine IL-15 mRNA was not detected in Meth-A cells transfected with IL-15 and vector alone (Figure 2). The production of IL-15 was confirmed by the bioassay of CTLL-2 proliferation. The level of IL-15 production in the transfectants ($10^6$ cells 24 $h^{-1}$) was 35 pg, 53 pg, and 42 pg for clones 2, 14 and 16 respectively. The bioactivity of each culture supernatant was neutralized completely by anti-IL-15 antibody at a concentration of 10 μg $ml^{-1}$. The Meth-A cells transfected with vector alone (clones 19–22) and parental cells did not produce bioactive IL-15. IL-15 levels, measured by the parallel use of an ELISA kit, were 25 pg, 31 pg and 29 pg for clone 2, 14 and 16 respectively. Moreover, the production of IL-15 by these three clones was stable for more than 1 year, because the fluctuations of this production at different times were < 15% in each clone. IL-15 production could not be detected in the supernatant of the parental cells or in transfectants by vector alone. We selected clones 16 (named Meth-A/IL-15), 2 (Meth-A/2), 14 (Meth-A/14) and 20 (Meth-A/Neo) for further examination.

*© 1999 Cancer Research Campaign* 

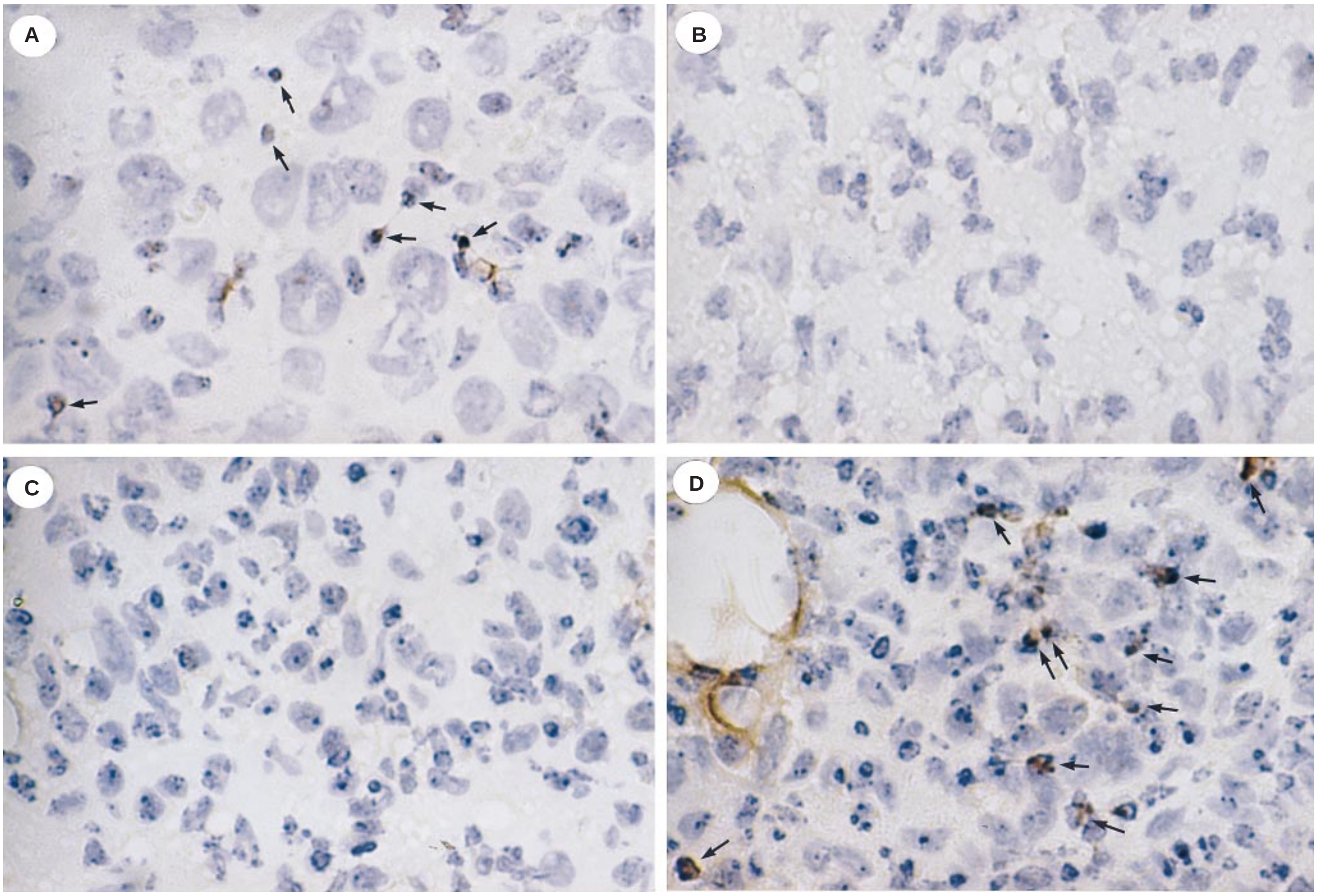

**Figure 5** Immunohistochemical analysis of tumour implantation sites in BALB/c mice 7 days after subcutaneous injection of Meth-A/IL-15 cells (**A**, **B**) and rechallenged parental cells (**C**, **D**). Staining with the L3/T4 (CD4) (**A**), KT15 (CD8) (**B**) revealed an infiltrate of $CD4^+$ T-cell in the Meth-A/IL-15 tumour. $CD8^+$ T-cells were virtually absent. In the rechallenged sites, there was a predominant infiltration of $CD8^+$ T-cells (**D**). $CD4^+$ T-cells (**C**) were virtually absent. Arrows indicate the positively stained cells

### Inhibition of tumour growth in vivo

The transfection of IL-15 or vector alone did not alter the growth properties of Meth-A cells in vitro as analysed by doubling time or morphology (data not shown). Tumorigenicity of Meth-A/IL-15 and Meth-A/Neo cells was examined by s.c. injection into BALB/c and BALB/c *nu/nu* mice in three independent experiments. In BALB/c mice Meth-A/IL-15 cells initially grew, then gradually regressed and were completely rejected within 21 days ($n$ = 10), while Meth-A/Neo cells grew progressively until they caused the death of the animals ($n$ = 10) (Figure 3A). There was no difference in the growth patterns of the three IL-15-secreting clones ($n$ = 5), Meth-A/IL-15, Meth-A/2 and Meth-A/14, of which production of IL-15 was 42 pg ($10^6$ cells 24 $h^{-1}$), 35 pg and 53 pg respectively (Figure 3C). Moreover, the growth of Meth-A/IL-15 ($n$ = 5) was augmented by the injection of anti-hIL-15 mAb (Figure 3D). In nude mice, both Meth-A/IL-15 cells ($n$ = 10) and Meth-A/Neo cells ($n$ = 10) grew progressively and were not rejected, although tumour growth was relatively inhibited in the case of Meth-A/IL-15 cells (Figure 3B).

### Histology at the site of tumour cell injection

Histological analysis of the injected site was performed at 7 and 14 days following the injection of tumour cells in order to characterize the host cellular responses augmented by IL-15 production. In BALB/c mice, massive infiltration of mononuclear cells and polymorphonuclear neutrophils was noted on day 7 after the implantation of Meth-A/IL-15 cells (Figure 4A). Moreover, on day 14, Meth-A/IL-15 cells were scattered and infiltrating cells were increased (Figure 4C). In contrast, in the case of Meth-A/Neo cells, few mononuclear infiltrates were observed (Figure 4B, D). In BALB/c nude mice, few mononuclear cells and neutrophils were observed at the injection sites of both Meth-A/IL-15 cells and Meth-A/Neo cells (Figure 4E, F).

Immunohistochemical analysis of tumour implantation sites in BALB/c mice 7 days after s.c. injection of Meth-A/IL-15 cells revealed a infiltrate of $CD4^+$ T cell (Figure 5A) and $CD11b^+$ cell (not shown) in the Meth-A/IL-15 tumour. $CD8^+$ T-cells (Figure 5B) were virtually absent.

### Rechallenging with parental Meth-A and colon-26 cells

We next examined whether the primary rejection of IL-15 transfectants leads to protective immunity. Thirty days after disappearance of the initial Meth-A/IL-15 implantation, 20 mice were injected with $2 \times 10^6$ parental Meth-A cells in the previously uninjected side (left) of the lower abdominal quadrant. In all mice, tumours were rejected by 21 days, though transient tumour growth was observed up to the 3rd day after injection (Figure 6A).

 © 1999 Cancer Research Campaign

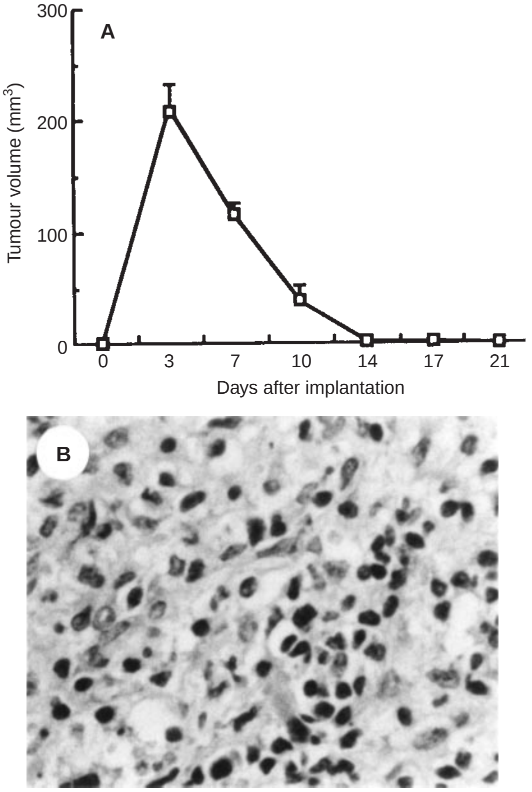

**Figure 6** In vivo growth of rechallenged parental Meth-A cells and histology of the rechallenged sites. (**A**) After disappearance of the initial Meth-A/IL-15 implantation, $2 \times 10^6$ parental Meth-A cells were injected into the opposite (left) lower abdominal quadrant. Tumour volumes are mean ± s.e.m. (**B**) Seven days after implantation the injection sites were resected, and paraffin sections were stained with haematoxylin and eosin (× 400)

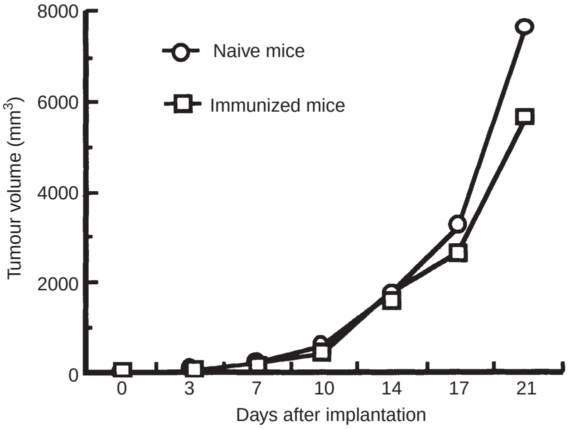

**Figure 7** In-vivo growth of initial-challenged or rechallenged parental Colon-26 cells. After disappearance of the initial Meth-A/IL-15 implantation, $5 \times 10^5$ Colon-26 cells were injected into the opposite (left) lower abdominal quadrant (*n* = 10). At the same time, Colon-26 cells were injected into non-immunized mice (*n* = 10). Tumour volumes are mean. Each s.e.m. was < 10%*

Interestingly, the remarkable infiltration of mononuclear cells without neutrophils at the tumour site was seen 7 days after implantation (Figure 6B).

Immunohistochemical analysis of rechallenged sites 7 days after s.c. injection of parental Meth-A cells revealed an infiltrate of $CD8^+$ T-cells (Figure 5D) and $CD11b^+$ cells (not shown) in the Meth-A/IL-15 tumour. $CD4^+$ T-cells (Figure 5C) were virtually absent.

To demonstrate the specificity of the protective immunity described above, Colon-26 cells, a murine colon adenocarcinoma derived from BALB/c mice, were injected into both non-immunized (*n* = 10) and immunized mice (*n* = 10). There was no significant difference in the growth of implanted Colon-26 cells between non-immunized mice and immunized mice (Figure 7).

### DISCUSSION

It is well established that transfected tumour cells, which secrete a broad variety of cytokines, are rejected due to the cytokine-induced increase in the host's anti-tumour defense (Colombo and Forni, 1994). These effects provide new experimental approaches for cancer immunotherapy. Namely, tumour cell-targeted cytokine gene therapy is achieved by the transfer and expression of appropriate genes in tumour cells in vitro followed by analysis of their growth characteristics in vivo. Here we report that such tumour rejection can be obtained by local IL-15 production.

Different mechanisms have been demonstrated in cells transfected with different cytokines. IFN-γ, IL-2, IL-6 and IL-12 transfectants have induced mainly specific CTL activity (Colombo and Forni, 1994; Zitvogel et al, 1995), while TNF-α, IL-4, MIP-1α and IL-8 induced non-specific anti-tumour activity and granulocyte infiltration (Colombo and Forni, 1994; Hirose et al, 1995). A new cytokine, IL-15, has shown many functional similarities to IL-2, such as stimulating proliferation of T- and B-lymphocytes and NK cells (Grabstein et al, 1994; Armitage et al, 1995; Munger et al, 1995; Mori et al, 1996). These properties suggest that IL-15, like IL-2, may have a role in cancer treatment. However, differences in the expression pattern of IL-15 and its receptor compared to the IL-2/IL-2R system also suggest unique in vivo roles for IL-15 (Andeson et al, 1995; Giri et al, 1995; Bamford et al, 1996). The present study is the first demonstration of the modification of host immune responses due to the implantation of syngeneic tumour cells engineered to secrete IL-15.

This study provides two new observations. First, we demonstrated that hIL-15-transfected tumour cells were rejected completely in syngeneic mice, but not in nude mice. This rejection was accompanied by predominantly a mononuclear ($CD4^+$ T-lymphocyte) and neutrophilic infiltration. It is possible that hIL-15 expressed by the tumour cells may be recognized as foreign and function as a tumour-rejection antigen for murine T-cells. However, we observed that the growth of Meth-A/IL-15 was augmented by anti-IL-15 injection. This result may support the direct effects of IL-15 on T-lymphocytes. Second, rechallenged parental cells were also rejected completely. This was associated with a predominantly mononuclear cell ($CD8^+$ T-lymphocyte) infiltration, but not a neutrophilic infiltration.

In spite of these demonstrated anti-tumour effects of IL-15 in vivo, our understanding of the underlying mechanism is rudimentary. IL-15 has been reported to activate and stimulate proliferation of T- and B-lymphocytes in vitro (Grabstein et al, 1994; Armitage et al, 1995; Mori et al, 1996). It stimulates the proliferation of NK-cells and acts as a co-stimulator with IL-12 to facilitate the synthesis of IFN-γ and TNF-α (Carson et al, 1996). IL-15 also facilitates the induction of cytolytic effecter cells, including CTL and LAK cells (Grabstein et al, 1994; Gamero et al, 1995; Lewko et al, 1995; Munger et al, 1995). In general, it is well-established that tumour rejection induced by local secretion of cytokines is mediated by a dual mechanism involving specific and non-specific anti-tumour effectors. With regard to the specific effects, our results indicate that IL-15 stimulates in vivo the sensitization of memory T-lymphocytes which are responsible for the specific long-lasting anti-tumour protection. Specifically, BALB/c mice that rejected Meth-A/IL-15 cells were protected against parental Meth-A tumours associated with $CD8^+$ T-lymphocytic infiltration, but not against Colon-26 tumours. Direct effects of IL-15 on T-lymphocytes seems likely since the IL-15 stimulation of T-lymphocytes may directly utilize the trimolecular IL-15 receptor complex (Giri et al, 1994, 1995; Grabstein et al, 1994; Andeson et al, 1995). Interestingly, massive neutrophilic infiltration was observed in tumour sites of BALB/c mice that rejected Meth-A/IL-15 cells, though it has been reported that IL-15 does not attract neutrophils (Wilkinson et al, 1995). These reports suggest that the neutrophilic infiltration may be due to a cascade of cytokines and soluble mediators, which were produced by activated T lymphocytes and other cells induced by the IL-15 secretion. Cavallo et al (1992) have reported that rejection of IL-2-transfected tumours is associated with neutrophil infiltration, and this neutrophil-dominated rejection generates a long-lasting, tumour-specific, T-lymphocyte-mediated immune memory. The role of neutrophils remains to be characterized in our studies.

The amounts of IL-15 secreted may be important. Meazza et al (1997) showed the poor efficiency of IL-15 natural signal peptides. They described that substitution of the sequence encoding natural signal peptide(s) with the one from IgVκ chain in the IL-15 cDNA resulted in a significantly higher secretion of biologically active IL-15 (15- to 30-fold) upon cDNA transfection. Although the amount of IL-15 produced by our transduced cells was relatively low, the in vitro effects were obtained.

In conclusion, these studies demonstrate that IL-15-transfected cancer cells can elicit an anti-tumour immune response and suggest that IL-15 may have a role as a component of tumour vaccines. Further studies are needed to understand the role of lymphocytes and neutrophils in the response to IL-15.

### ACKNOWLEDGEMENTS

This work was supported in part by Grants-in-Aid from the Ministry of Education, Science, Sports and Culture of Japan (project No. 08877201 and 09470268).

### REFERENCES

Andeson DM, Kumaki S, Ahdieh M, Bertles J, Tometsko M, Loomis A, Giri JG, Copeland NG, Gilbert D, Jenkins NA, Valentin V, Shapiro DN, Morris SW, Park LS and Cosman D (1995) Functional characterization of the human interleukin-15 receptor α chain and close linkage of IL15RA and IL2RA genes. *J Biol Chem* **270**: 29862–29869

Armitage RJ, Macduff BM, Eisenman J, Paxton R and Grabstein KH (1995) IL-15 has stimulatory activity for the induction of B cell proliferation and differentiation. *J Immunol* **154**: 483–490

Bamford RN, Battiata AP and Waldmann TA (1996) IL-15: the role of translational regulation in their expression. *J Leukocyte Biol* **59**: 476–480

Carson WE, Giri JG, Lindemann MJ, Linett ML, Ahdieh M, Paxton R, Anderson D, Eisenmann J, Grabstein K and Caligiuri MA (1994) Interleukin (IL) 15 is a novel cytokine that activates human natural killer cells via components of the IL-2 receptor. *J Exp Med* **180**: 1395–1403

Cavallo F, Giovarelli M, Gulino A, Vacca A, Stoppacciaro A, Modesti A and Forni G (1992) Role of neutrophils and $CD4^+$ T lymphocytes in the primary and memory response to nonimmunogenic murine mammary adenocarcinoma made immunogenic by IL-2 gene. *J Immunol* **149**: 3627–3635

Colombo MP and Forni G (1994) Cytokine gene transfer in tumor inhibition and tumor therapy: where are we now? *Immunol Today* **15**: 48–51

Corbett TH, Griswold DP Jr, Roberts BJ, Peckham JC and Schabel FM Jr (1975) Tumor induction relationships in development of transplantable cancers of the colon in mice for chemotherapy assay, with a note on carcinogen structure. *Cancer Res* **35**: 2434–2439

DeLeo AB, Shiku H, Takahashi T, John M and Old LY (1977) Cell surface antigens of chemically induced sarcomas of the mouse. I. Murine leukemia virus-related antigens and allo-antigens on cultured fibroblasts and sarcoma cells: description of a unique antigen on BALB/c Meth A sarcoma. *J Exp Med* **146**: 720–734

Gamero AM, Ussery D, Reintgen DS, Puleo CA and Djeu JY (1995) Interleukin 15 induction of lymphokine-activated killer cell function against autologous tumor cells in melanoma patient lymphocytes by a CD18-dependent, perforin-related mechanism. *Cancer Res* **55**: 4988–4994

Giri JG, Ahdieh M, Eisenman J, Shanebeck K, Grabstein K, Kumaki S, Namen A, Park LS, Cosman D and Anderson D (1994) Utilization of the β- and γ-chains of the IL-2 receptor by the novel cytokine IL-15. *EMBO J* **13**: 2822–2830

Giri JG, Kumaki S, Ahdieh M, Friend DJ, Loomis A, Shanebeck K, DuBose R, Cosman D, Park LS and Anderson DM (1995) Identification and cloning of a novel IL-15 binding protein that is structurally related to the α chain of the IL-2 receptor. *EMBO J* **14**: 3654–3663

Grabstein KH, Eisenman J, Shanebeck K, Rauch C, Srinivasan S, Fung V, Beers C, Richardson J, Schoenborn MA, Ahdieh M, Johnson L, Alderson MR, Watson JD, Anderson DM and Giri JG (1994) Cloning of a T cell growth factor that interacts with the β-chain of the interleukin-2 receptor. *Science* **264**: 965–968

Hirose K, Hakozaki M, Nyunoya Y, Kobayashi Y, matsushita K, Takenouchi T, Mikata A, Mukaida N and Matsushima K (1995) Chemokine gene transfection into tumor cells reduced tumorigenicity in nude mice in association with neutrophilic infiltration. *Br J Cancer* **72**: 708–713

Iizuka N, Oka M, Noma T, Nakazawa A, Hirose K and Suzuki T (1995) NM23-H1 and NM23-H2 messenger RNA abundance in human hepatocellular carcinoma. *Cancer Res* **55**: 652–657

Lebel-Binay S, Laguerre B, Quintin-Colonna F, Conjeaud H, Magazin M, Miloux B, Pecceu F, Caput D, Ferrara P and Fradelizi D (1995) Experimental gene therapy of cancer using tumor cells engineered to secrete interleukin-13. *Eur J Immunol* **25**: 2340–2348

Lewko WM, Smith TL, Bowman DJ, Good RW and Oldham RK (1995) Interleukin-15 and the growth of tumor derived activated T cells. *Cancer Biother* **10**: 13–20

Meazza R, Gaggero A, Neglia F, Basso S, Sforzini S, Pereno R, Azzarone B and Ferrini A (1997) Expression of two interleukin-15 mRNA isoforms in human tumors does not correlate with secretion: role of different signal peptides. *Eur J Immunol* **27**: 1049–1054

Mori A, Suko M, Kaminuma O, Inoue S, Ohmura T, Nishizaki Y, Nagahori T, Asakura Y, Hoshino A, Okumura Y, Sato G, Ito K and Okudaira H (1996) IL-15 promotes cytokine production of human T helper cells. *J Immunol* **156**: 2400–2405

Munger W, DeJoy SQ, Jeyaseelan R Sr, Torley LW, Grabstein KH, Eisenmann J, Paxtox R, Cox RT, Wick MM and Kerwar SS (1995) Studies evaluating the antitumor activity and toxicity of interleukin-15, a new T cell growth factor: comparison with interleukin-2. *Cell Immunol* **165**: 289–293

Nishimura H, Washizu J, Nakamura N, Enomoto A, and Yoshikai Y (1998) Translational efficiency is up-regulated by alternative exon in murine IL-15 mRNA. *J Immunol* **160**: 936–942

Tokunaga K, Taniguchi H, Yoda K, Shimizu M and Sakiyama S (1986) Nucleotide sequence of a full-length cDNA for mouse cytoskeletal beta-actin mRNA. *Nucleic Acids Res* **14**: 2829–2829

Workman P, Balmain A, Hickman JA, McNally NJ, Mitchison, Pierrepoint CG, Raymond R, Rowlatt C, Stephens TC and Wallace J (1988) UKCCCR *Guidelines for the Welfare of Animals in Experimental Neoplasia*. *Br J Cancer* **58**: 109–113

Wilkinson PC and Liew FY (1995) Chemoattraction of human blood T lymphocytes by interleukin-15. *J Exp Med* **181**: 1255–1259

Zitvogel L, Tahara H, Robbins PD, Storkus WJ, Clarke MR, Nalesnik MA and Lotze MT (1995) Cancer immunotherapy of established tumors with IL-12. Effective delivery by genetically engineered fibroblasts. *J Immunol* **155**: 1393–1403

 © 1999 Cancer Research Campaign
